# A Dynamic Spatio-Temporal Model to Investigate the Effect of Cattle Movements on the Spread of Bluetongue BTV-8 in Belgium

**DOI:** 10.1371/journal.pone.0078591

**Published:** 2013-11-11

**Authors:** Chellafe Ensoy, Marc Aerts, Sarah Welby, Yves Van der Stede, Christel Faes

**Affiliations:** 1 Interuniversity Institute for Biostatistics and statistical Bioinformatics, Universiteit Hasselt, Hasselt, Belgium; 2 Veterinary and Agrochemical Research Centre, Brussels, Belgium; 3 University of Ghent, Laboratory of Vet. Immunology, Merelbeke, Belgium; National University of Singapore, Singapore

## Abstract

When Bluetongue Virus Serotype 8 (BTV-8) was first detected in Northern Europe in 2006, several guidelines were immediately put into place with the goal to protect farms and stop the spreading of the disease. This however did not prevent further rapid spread of BTV-8 across Northern Europe. Using information on the 2006 Bluetongue outbreak in cattle farms in Belgium, a spatio-temporal transmission model was formulated. The model quantifies the local transmission of the disease between farms within a municipality, the short-distance transmission between farms across neighbouring municipalities and the transmission as a result of cattle movement. Different municipality-level covariates such as farm density, land composition variables, temperature and precipitation, were assessed as possibly influencing each component of the transmission process. Results showed a significant influence of the different covariates in each model component, particularly the significant effect of temperature and precipitation values in the number of infected farms. The model which allowed us to predict the dynamic spreading of BTV for different movement restriction scenarios, also affirmed the significant impact of cattle movement in the 2006 BTV outbreak pattern. Simulation results further showed the importance of considering the size of restriction zones in the formulation of guidelines for animal infectious diseases.

## Introduction

For decades, the livestock industry has been battling the emergence and recurrence of various infectious animal diseases. The negative social, economic and environmental impact brought on by these diseases is a major concern not only for this industry, but also for the countries involved and the international community [1]. Prevention and control measures are then usually prepared at the national and international level. One of the important control measures is the restriction of animal movement between farms and/or countries during an outbreak [2]. This control measure comes from the knowledge that movement provides an important route of transmission of infectious diseases [3], [4], [5]. As in the case of the foot-and-mouth disease (FMD) epidemic in 2001, Févre et al. [3] reported that the spread of FMD from the north of England to France and the Netherlands was due to animal movement. Other animal diseases also have the potential to be spread through animal movements, such as rabies [3], bovine tuberculosis [3], Coxiellosis [5] and bluetongue [6].

Bluetongue (BT) is a non-contagious, infectious, vector-borne disease of ruminants caused by the bluetongue virus (BTV) and is transmitted between hosts by bites of *Culicoides* midges. Over the past decade BT has become one of the most important diseases of livestock following a series of incursions in Europe [7]. In particular, the first cases of BTV serotype 8 (BTV-8) in northern Europe were reported near Maastricht in the Netherlands in July/August 2006, with subsequent cases reported in Belgium, Germany, France and Luxembourg. In May 2007, BTV-8 re-emerged and caused major outbreaks across the previously-affected countries and spread into new areas [8].

Since the time it was detected up to the present, several studies have been conducted which explained why and how the BTV outbreak occurred. Some of these studies looked into the risk factors associated with BTV such as climatic conditions, land composition [9],[10],[11], while others looked in greater detail on the effect of animal movement on the spread of the virus [6], [12], [13], [14]. In this study, a spatio-temporal transmission model was formulated using data of the 2006 BTV outbreak in cattle farms in Belgium. The proposed model quantifies the local transmission of the disease between farms within a municipality, the transmission between farms across neighbouring municipalities and transmission as a result of the movement/transport of animals. Municipality-level factors influencing the transmission process were also investigated.

In the subsequent section, an overview of the BTV-8 outbreak, risk-factor and cattle movement data are given. This is then followed by a detailed description of the proposed spatio-temporal transmission model and the procedure for model selection. Results of the model fitting are then presented along with simulation results followed by a brief discussion.

## Materials and Methods

### Data Sources

#### Outbreak Data

**Figure 1:**
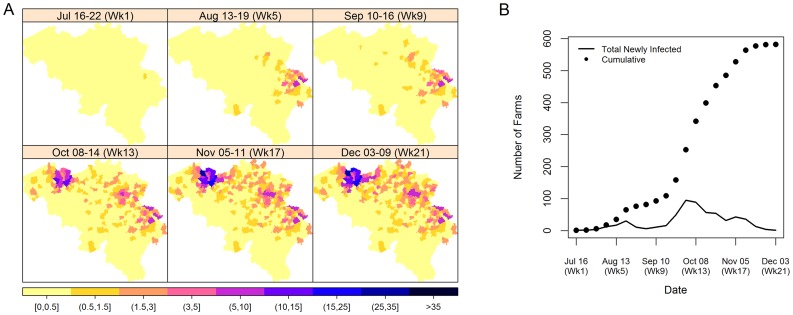
Spatial and temporal trend of the BTV-8 outbreak in Belgium for 2006. Figures are based on the weekly data with (A) giving the spatial trend of the cumulative number of infected farms and (B) giving the temporal trend of the weekly new infections and cumulative number of infected farms. The onset of infection was the date that disease symptoms were first observed, assumed 3 to 4 weeks before confirmation of report.

**Figure 2:**
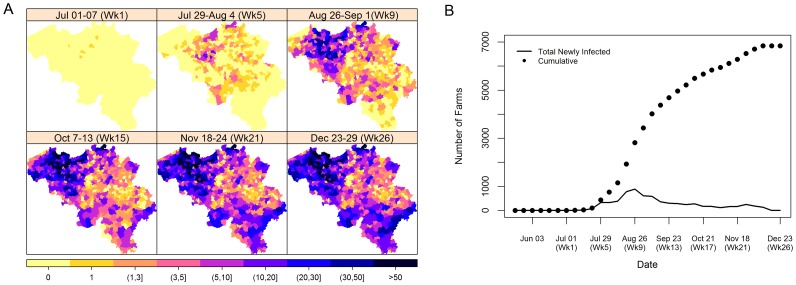
Spatial and temporal trend of the BTV-8 outbreak in Belgium for 2007. Figures are based on the weekly data with (A) giving the spatial trend of the cumulative number of infected farms and (B) giving the temporal trend of the weekly new infections and cumulative number of infected farms.

The 2006 Bluetongue outbreak information for cattle in Belgium used in this study was obtained from the Veterinary and Agrochemical Research Centre (CODA-CERVA), Belgium. Figures 1 and 2 detail the observed spatial and temporal trend of the BTV-8 outbreak in Belgium for 2006 and 2007, respectively. Farms having at least 1 observed infected animal were considered as infected. The onset of infection that was used in this study was the date that the infection was thought to have occurred similar to the dates used by Faes et al. [10]. We have used the date that the disease symptoms were first observed as the date of infection as we have no knowledge on when the infection actually occurred.

#### Risk Factor Data

**Figure 3:**
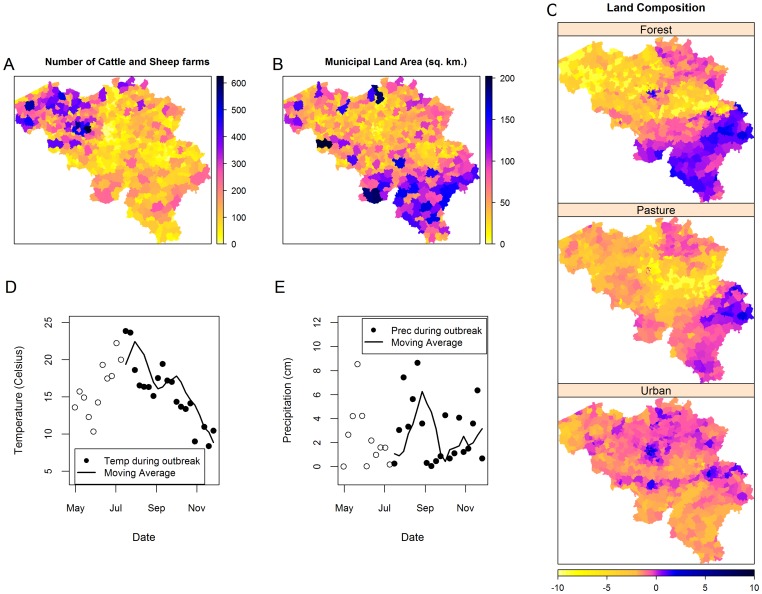
Risk factor data. (A) Number of cattle and sheep farms; (B) Land area per municipality (in square km); (C) Land composition variables with respect to proportion of crop land; (D) and (E): Average weekly temperature and precipitation in Belgium during the 2006 outbreak. With the moving average computed as the average of the lag 1 to 4 weeks values.

Different covariates deemed influential to the spread of BTV [15], [12], [10] were investigated. These risk factors (Figure 3) include:

- Farm and animal (cattle and sheep) density per municipality,
- Proportion of forest, crop, urban, and pasture area per municipality,
- Temperature, and
- Precipitation.

Land use variables (proportion of forest, crop, urban, and pasture area per municipality) are highly correlated owing to the fact that each of the variables convey relative information to the whole. To deal with this issue, these covariates were transformed using the compositional data technique based on the additive log-ratio [16]. The additive log-ratio transformation works by taking the log of the ratio of a covariate and another reference covariate. Applying this transformation to the compositional data resulted in less correlated variables with values which can vary over the entire real number range. In this study, the crop variable was taken as the reference and the other variables were transformed into: 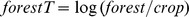, 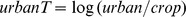 and 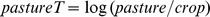. The problem of zero value for the crop proportion was handled by adding a small constant (0.0001) to all variables. Only the transformed forest, urban, and pasture variables were entered into the model, and all of them were interpreted in terms of the proportion of crop land.

For the temperature and precipitation, it has been shown that seasonal variations in weather affect the spread of *Culicoides* and therefore also affect the spread of BTV [17], [9], [11]. Temperature and precipitation data (daily mean temperature (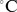) and precipitation (cm)) from all weather station of Belgium were obtained and then summarized to average weekly readings (black circles in Figure 3D and 3E with black solid dots signifying values during the outbreak period). Since Belgium is a very small country (total area of 30,528 sq. km.), the temperature and precipitation reading were observed not to differ much from one weather station to the next and hence assumed to be also the same in all non-station locations. Readings from all weather stations were aggregated to average weekly readings and both variables were assumed constant throughout the whole country. Based on results from various studies, [11], [15], [18], a moving average of these values at time lag of 1 to 4 weeks (black solid line in the figure) was used to ensure a smooth trend and to consider the time needed for the vector population to develop a competent population, and to account for the uncertainty of the date of the infectious bite.

#### Transport of Animals

Purchase of an animal results in movement of the animal from one farm to the other. If the farm of origin has cases of BT infection, there is a certain probability that the animal was also infected thereby increasing the chance that the animals in the destination farm will also, via vectors, be infected (see [6] and [14]). To account for this source of infection, the number of animal movements across different municipalities was explored. Animal movement in this paper refer to cattle movement only. Although sheep movement might also be an important source of infection, it was not included in this paper as there is no available information for sheep movement in Belgium. Furthermore, in Belgium, sheep are raised for meat and breeding as a hobby [19], hence movement concern only ovines of high genetic performance, between large or high producer ovine herds, which constitutes only a minority of the sheep herds in Belgium.

Movement information was extracted from the cattle birth and purchase information in Belgium. Birth data were however only available from 2005, thereby limiting the number of cattle movement which could be traced. It was thus decided to use a constant general pattern of movement based on the 2005 to 2009 data given the fact that a rather similar trend of purchasing over the years was observed (Figure S1).

Defining cattle movement as the farm-to-farm transfer of cattle through purchasing, the number of animal movements for two different municipalities was counted. Thus, movement of cattle is defined if cattle were transferred/transported/purchased from a farm in municipality 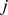 to a farm in municipality 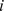. Due to the restriction on the resolution of the data, transport between farms in the same municipality was not considered as movement in this case. Two ways of quantifying the movement were explored:

- Presence of movement (Binary indicator of transport, taking the value of 1 if at least one animal is transported and 0 otherwise),
- Relative movement which show the abundance of movement (Proportion of animals transported).

**Figure 4:**
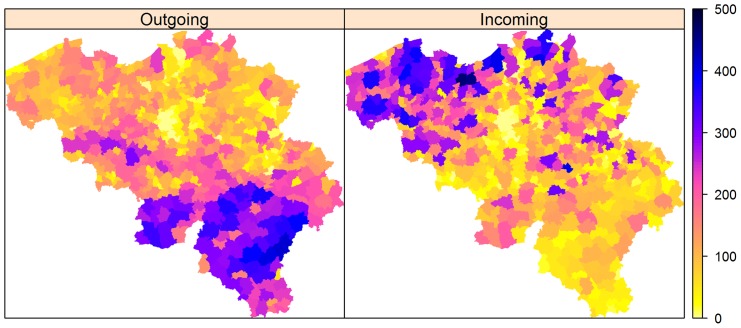
Spatial pattern of cattle movement in Belgium. The total number of outgoing and incoming cattle movement between municipalities obtained from the 2005–2009 cattle birth and purchase information in Belgium. A municipality (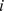) is defined to have an outgoing movement if there is a transport of cattle from that municipality (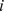) to municipality (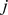). Municipality (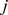) is then defined to have an incoming movement.

Figure 4 shows the general pattern of contacts derived from the binary definition of transport and shows that most movements originate from the Walloon area and end up in the Flemish region, and in particular, in the provinces of Antwerp, East and West Flanders.

### Infection Model

Mathematical modeling of an infectious disease is a tool used to describe the dynamics of the spread of that infectious disease and can be used to evaluate different control strategies (e.g. movement restriction). One of the most common models used is the SIR (Susceptible 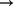 Infected 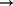 Recovered) model. The SIR model postulates that an individual or unit starts in the susceptible class, can become infected by the disease, thus moving to the infected class, and after a while, recover from it. Different variations of this mathematical model are available (e.g. SIS, SIRS and SEIS (Susceptible 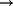 Exposed 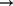 Infected 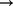 Susceptible)), depending on the disease in question [20].

In the case of BTV, a modified SEI and SEIR model can be found in the literature [7], [9], [14]. In this paper, using the farm as an individual unit and assuming that once infected, a farm is infectious until the end of the outbreak, a SI model was applied. The assumption of no recovery of infected farms within the outbreak period was based on the long recovery time of an infected cattle, with BTV-8 virus still detected in cattle 1–2 months after infection [21], [22], [23].

Each farm was classified into either susceptible **S** (no cattle infected with BTV), or infected and infectious **I** (at least one reported case of infected cattle). An infectious farm can then infect another farm through the vector (*Culicoides* midges). Although it would have been preferable to build an individual cattle-based model rather than a farm-based model, the reporting procedure (owners report only the first observed infection) constrained the analysis to farm level. Furthermore, the unavailability of the vector data constrained the analysis to a basic SI model.

The SI model for BT is a closed population model, where 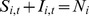, and 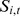 and 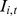 are the number of susceptible and infectious farms respectively, in week 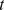 at municipality 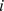 and 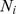 is the total number of farms for each municipality 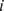. The number of susceptible farms can then be written as the difference of the total number of farms and the number of infectious farms, while the number of infectious farms at week 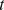 is just the sum of the total number of newly infected farms (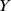) until week 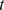 and is given by:
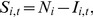
(1)

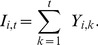
(2)


Several authors have proposed various ways of modelling the number of infected farms. Held et al.[24] proposed a Poisson branching process model, Knorr-Held and Richardson [25] used a hierarchical hidden Markov model, while Schrödle et al. [5] used parameter-driven and observation-driven models to link the movement and spreading of diseases. In this study, the number of newly infected farms (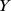) was modelled as a binomial random variable which depends on the number of susceptible farms at the previous week (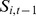) and a parameter 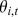, 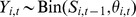. The parameter 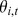 was formulated as a function of the previous infectious population via the following equation similar to the method by Hooten et al. [26]:
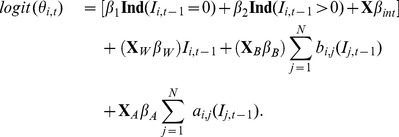
(3)


This dynamic infection model contains at most four additive terms representing the different transmission scenarios. The first term, 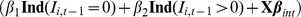 represents the background risk of the municipality which depends on municipality-specific covariates and where the overall risk is increased or decreased depending on whether or not there was an infectious farm in the municipality at the previous time. Similarly, the second term 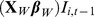 is only present when an infection was observed previously, and corresponds to the within-municipality transmission or the local spread. This term expresses the belief that the number of infected farms in municipality 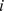 at the current week is a function of the number of infectious farms at the previous week and some covariates (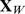). The coefficient 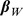 represents the contribution of each covariate to the local transmission.

The third term, 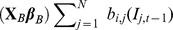, deals with the neighbourhood or between-municipality transmission, representing the effect of the infectious state of neighbouring municipalities the previous week, together with some municipality-level covariates. The spatial weight 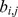 was derived based on contiguity between municipalities 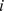 and 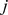. Municipalities considered as neighbours take the value of 1, otherwise they take the value of 0. This results in a symmetric weight matrix with a 0 diagonal. The binary weighting then ensures that a municipality with more infected neighbours is given more weight in the transmission model [27].

The final term 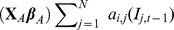 corresponds to long-distance transmission through animal movements. The 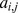 in this equation quantifies the movement of animals from municipality 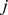 to municipality 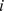 and is defined as explained above. Unlike the neighbourhood matrix, the movement weight matrix is asymmetric. This is based on the fact that the number of transports from municipality 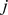 to 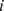 can be different from municipality 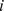 to 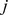. To ensure that only the long-distance transmission is reflected in this part of the model, local (movement between farms within the same municipality) and neighbourhood movements were taken out of the weight matrix (since the local and neighbourhood effect are already accounted for by the second and third term of the model). Similar to the second and third term, different environmental factors were included in this component. The argument behind this is that movement of infected cattle alone does not ensure transmission, it is the combination of movement and presence of vectors in the area.

### Model Selection and Exploration

Using the average weekly temperature, precipitation and their interaction, transformed pasture, forest and urban areas, farm density and total land area as covariates, equation (3) was fitted using Proc NLMIXED in SAS, version 9.2 (SAS Institute, Cary NC). Various representations (binary indicator, actual count, log-transformed count) of the infection (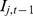) and movement (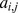) status in the model were explored. Model selection was done using the Akaike information criterion (AIC) with the model having the smallest AIC value selected as the best model. Based on the AIC values given in Table 1, the best fitting model was the model with a binary indicator for infection status (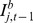) in the neighbourhood and movement components along with a binary movement weight (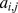).

**Table 1:** Comparison of the AIC for different model component choices.

Infection status ( 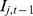 )	Movement ( 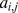 )		
in Component 3 and 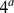	Binary	Count	Log-Count
Binary	3435.1	3444.2	3443.9
Count	3576.3	3594.6	3594.5

The spatio-temporal model is fitted to the 2006 BTV-8 outbreak data.

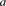
 Infection status in neighbourhood and movement components.

Some of the parameters in the full model were not significantly different from zero and hence the model could be reduced. Model reduction using the AIC criterion resulted in the retention of 24 parameters from the 37 parameters in the full model, and the AIC value was decreased by 15.5 (from 3435.1 to 3419.6). Parameter estimates for this reduced model are given in Table 2.

**Table 2:** Parameter Estimates for the reduced spatio-temporal model fitted to the 2006 BTV-8 outbreak data.

Component		Covariate	Estimate	95% CI
Background	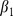	Intercept 1	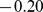	[ 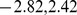 ]
	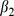	Intercept 2	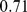	[ 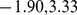 ]
	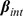	Temperature ( 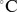 )	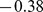	[ 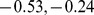 ]
		Precipitation (cm)	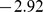	[ 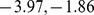 ]
		Temp x Prec	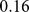	[ 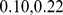 ]
		PastureT 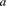	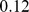	[ 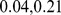 ]
		UrbanT 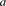	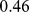	[ 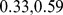 ]
		Farm Density 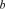	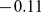	[ 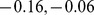 ]
		Land Area (sq. km.)	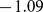	[ 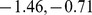 ]
Within-municipality	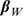	Intercept	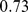	[ 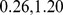 ]
		Temperature ( 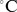 )	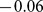	[ 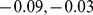 ]
		Precipitation (cm)	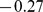	[ 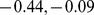 ]
		Temp x Prec	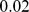	[ 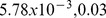 ]
		UrbanT 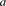	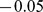	[ 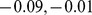 ]
		Land Area (sq. km.)	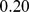	[ 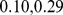 ]
Between-municipality	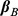	Intercept	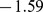	[ 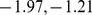 ]
		Temperature ( 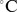 )	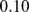	[ 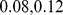 ]
		Precipitation (cm)	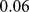	[ 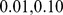 ]
		PastureT 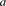	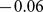	[ 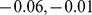 ]
		UrbanT 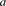	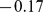	[ 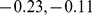 ]
Movement	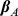	Intercept	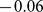	[ 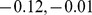 ]
		Temperature ( 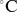 )	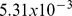	[ 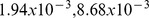 ]
		Precipitation (cm)	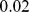	[ 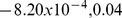 ]
		Temp x Prec	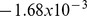	[ 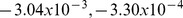 ]

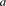
 Log-ratio transformed 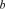 Number of cattle and sheep farms per municipality.

## Results

Figure 1 shows that, in 2006 BTV-8 in Belgium first appeared around the area of Liège during the 3rd week of July (considered as week 1 of the outbreak). The infection then spread within Liège and around Limburg and neighbouring provinces and reached its peak during the 6th week of the outbreak (August 20–26). It was also during this week that the whole country of Belgium was declared as BTV-8 infected and thus movement restriction were lifted [13]. After this peak, a dying-out phase was observed with the total outbreak size of 82 farms during the 8th week. However, in the week of September 10–16 (week 9), a jump to the East Flanders area was observed, with the first case appearing in the municipality of Destelbergen, a neighbouring municipality of Ghent. It then quickly spread to other municipalities in the province (i.e. Ghent, Nevele and Deinze) during the succeeding weeks. By the end of 2006, out of the 40 141 cattle farms in Belgium (partitioned across 576 municipalities), a total of 582 cases of infected farms from 205 different municipalities was observed.

During the winter period, many hoped that BTV-8 had disappeared [28]. However, BTV-8 re-emerged in the first week of July 2007 and by the end of that year, 6 840 farms (9.5% of the total) across 90% of the municipalities, had notified an infection. This second episode was much larger than the one in 2006. It also involved areas which were previously not affected by the disease, notably municipalities in the southern part of Belgium.

**Figure 5:**
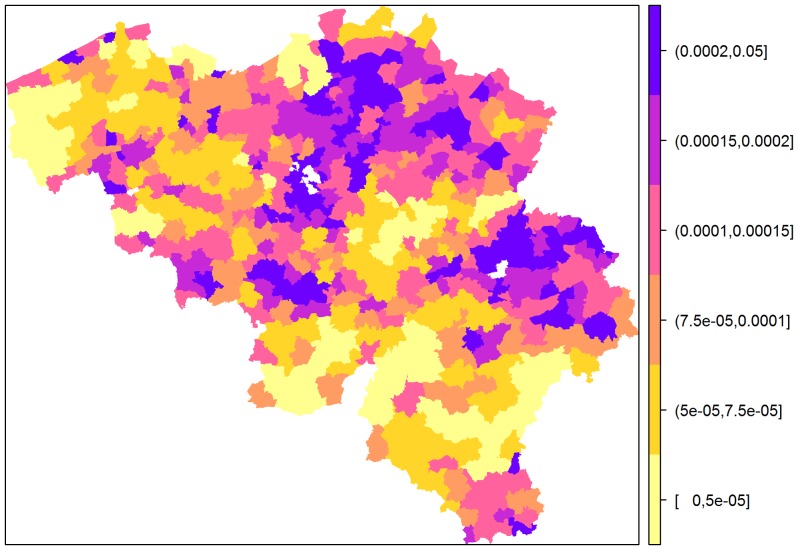
Spatial structure of the background odds in acquiring BTV. The map gives the odds of transmission during the start of the outbreak which is computed using 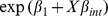.

Fitting the infection model to the data, results show that for any given farm in a municipality, the background odds of contracting BTV is increased by 2.50 (95% CI [1.89, 3.11]) if an infection had already occurred in that same municipality during the previous week. If infection was not observed in any municipalities at the previous time point, Figure 5 shows the inherent susceptibility of the different municipalities in acquiring BTV. The map for background transmission shows that depending on covariate values, some municipalities have higher odds of acquiring BT than others. Most areas with increased odds are found in Liège and in the provinces of Antwerp and Limburg, where infection was mostly observed.

**Figure 6:**
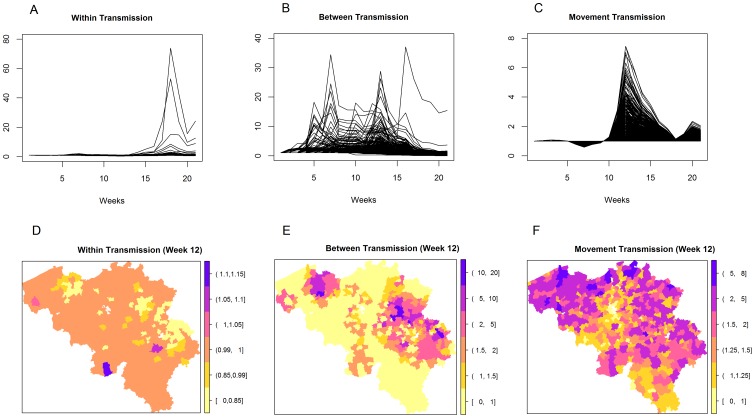
Odds of BT transmission within and between municipalities and through cattle movement. The top 3(A, B and C) gives the temporal trend of the contributions of each model component to the BT transmission while the maps at the bottom (D, E and F) gives the spatial structure of the contributions of each component during the peak of the outbreak (week 12 of 2006 outbreak, where week 1 is on 20–26 July). The odds were computed based on the within-municipality transmission, 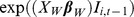; Between-municipality transmission, 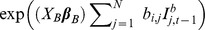; Movement transmission, 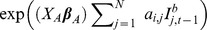.

Conversely, if infection was already observed in at least 1 municipality at the previous time point, the within-municipality, between-municipality and movement transmission comes into effect. Figure 6 (A, B and C) shows the temporal trend of within-municipality, between-municipality, and movement transmission contributions depending on the infection status at previous time points. The plots show a non-monotone change in transmission values, starting from 0 at week 1 and increasing or decreasing depending on the temperature and precipitation values at 1 to 4 weeks prior to the investigated time point. A smooth temporal pattern per municipality was furthermore observed for the movement transmission, coming from the fact that in the final model, no municipality-specific covariate for the movement transmission was retained, unlike in the within-municipality and between-municipality transmission. Table 2 shows that the within-municipality transmission was found to be significantly influenced not only by temperature, precipitation and their interaction, but also by the land area and proportion of urban area relative to crop area. The neighbourhood or between-municipality transmission on the other hand was significantly influenced by the proportion of pasture and urban areas relative to crop area, aside from temperature and precipitation.

Figure 6 (D, E and F) shows that during the second peak of the outbreak (12th week of outbreak in 2006), municipalities around Namur, Luxembourg and West Flanders have high odds of within-municipality transmission (Figure 6, D), while areas around Liège, Limburg and East Flanders have high odds of between-municipality transmission as compared to other areas (Figure 6, E). The maps clearly show that areas with low values for local transmission have high between-municipality transmission and vice versa. However, there are areas with high local and between-municipality transmission, although for these areas the odds of local transmission is slightly below or equal to 1. With regards to transmission through movements, Figure 6 (F) shows that areas with more incoming movements (Figure 4) have increased risk of BTV transmission. Hot spots were found in the provinces of Antwerp, East Flanders and Limburg. The pattern seen on the maps implies that during the peak of the 2006 outbreak, the spread of BTV was more due to the between-municipality and movement transmission rather than the within-municipality transmission.

### One-step-ahead and Long-term Prediction

**Figure 7:**
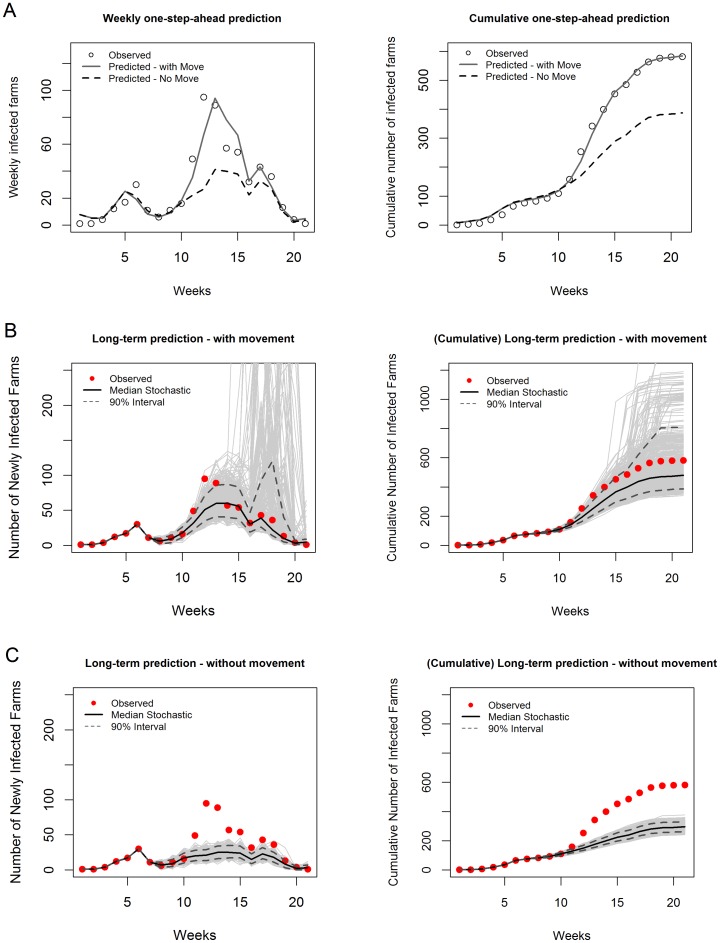
Predicted temporal trend of the 2006 outbreak. The weekly number of predicted infections and the cumulative number of infections are based on one-step-ahead (deterministic) predictions (A) and long-term (stochastic) predictions (B) with and (C) without cattle movements (complete ban). The gray lines are the predictions from 1000 simulations.

**Figure 8:**
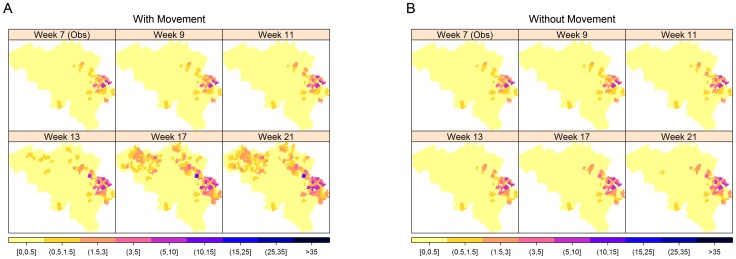
Predicted spatial trend of the 2006 outbreak. The maps show the median cumulative number of infected farms with (A) and without (B) animal movements based on 1000 stochastic simulations.

A one-step-ahead deterministic prediction and long-term stochastic prediction based on the parameters from the reduced model (Table 2) are depicted in Figure 7. The deterministic prediction (A) gives the current week predicted values based on the parameters of the reduced model (Table 2) and observed values of the previous week. The stochastic prediction (B and C), on the other hand, starts with an initial condition (e.g. introduction of one case in an area) and predicts future events by generating observations from a binomial distribution based on the predicted probabilities from the model. In this study, parameter estimates from the fitted model (Table 2) and data up until the 7th week of the outbreak (79 observed cases) were used as an initial condition and the model was then allowed to predict the rest of the outbreak period. The choice of the 7 weeks data coincided with the time (a week) after the lifting of the movement restriction. A total of 1000 simulations was done (gray lines in Figure 7) with the median stochastic prediction given by the black line. The model managed to capture fairly well both the temporal (Figure 7B) and spatial trend (Figure 8A) of the infection, with the true trend falling within the 90% interval.

To investigate the impact of movement of animals in the transmission of BTV, Figure 7 shows the one-step-ahead and long-term prediction from the model with and without movement restrictions. In the deterministic plot (A), complete absence of cattle movement throughout the outbreak duration was assumed, while for the stochastic prediction (C), complete movement restriction in the whole of Belgium was assumed to start from week 8 of the outbreak. We can see in the temporal plots the reduction of the predicted number of newly infected cases, and hence reduction in the number of cumulative infections per municipality when there is complete movement restriction (see Figure 8).

The median stochastic final size without animal movement was estimated to be 295 farms with a 90% confidence interval of 262–330 farms. This was significantly lower than the true outbreak final size of 582 farms. If there were no restriction, on the other hand, the median stochastic final size was estimated to be 480 farms with a 90% confidence interval of 387–810 farms. This reduction in the predicted number of cases suggests that animal movements have a significant impact on the spread of BTV. It can also be observed from the figures that with complete movement restriction, the noticeable jump in the Ghent region was not predicted by the model. This implies that the outbreak becomes limited only to the surrounding municipalities and provinces and the long-distance transmission of the virus does not occur with movement restrictions.

**Figure 9:**
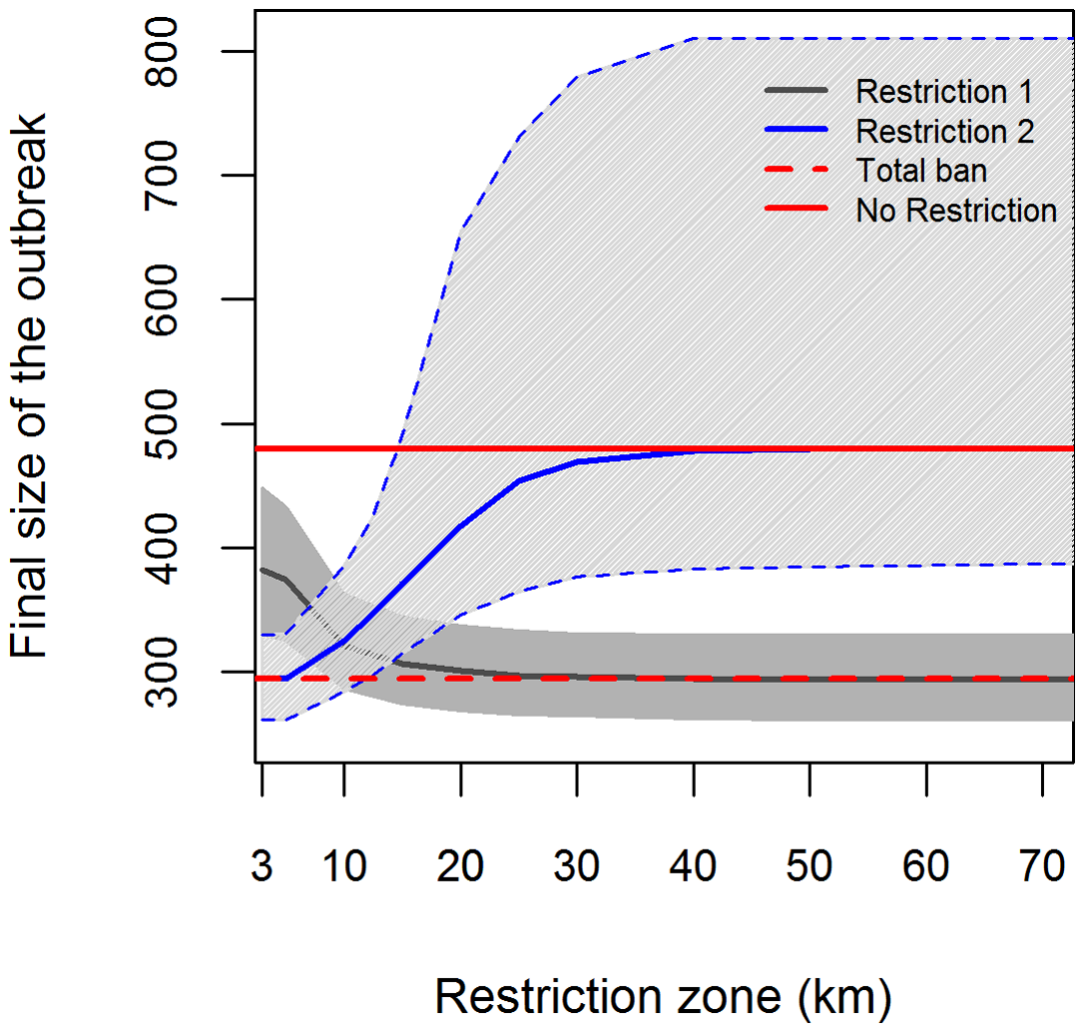
Final outbreak size as a function of movement restriction radius. Restriction 1 denotes movement restriction within the zone, while in restriction 2, movement within the zone is allowed. Values were based on 1000 stochastic simulations from the reduced spatio-temporal model. Data until week 6 of the 2006 outbreak was used and the model was allowed to predict the rest of the outbreak period.

To further investigate the effect of different restriction scenarios on the spread of BT, a stochastic prediction was simulated for 2 different types of restriction established within a certain radius (e.g. 20 km) around an infected farm. Restriction 1 denotes movement restriction within the zone, while in restriction 2, movement within the zone is allowed and only movement outside the zone is prohibited. In other words, when movement restriction 1 is in place, no movement of cattle is allowed within the restriction zone and from the restriction zone to outside the restriction zone, but for the rest of the country, cattle movement is allowed. Figure 9 shows that restricting the movement resulted in a significant reduction in the predicted final size of the outbreak, although it is apparent in the plot that this depends on both the type of restriction and the size of the restriction zone. For restriction 1, a 15 km restriction zone is already as effective as a total ban of movement and increasing the radius of the zone no longer leads to significant decrease in the outbreak size. Based on the bootstrap confidence interval however, a 10 km zone seems to be already sufficient. Restriction 2 on the other hand, is only effective up to around 10 km, increasing the restriction zone further also increases the predicted outbreak size. It should be noted that the restriction zone which was set-up during the start of the 2006 epidemic and lifted during August was similar to the second type of restriction investigated here and covers a radius of 20 km.

### Application to the 2007 Outbreak

**Figure 10:**
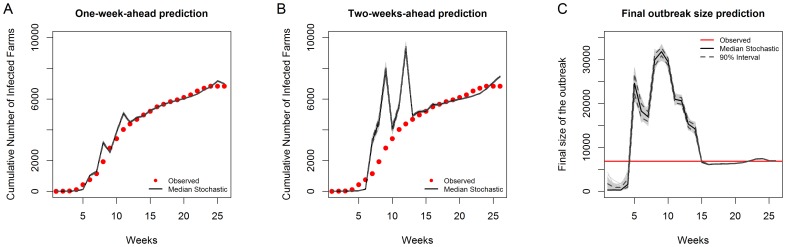
One-week and two-weeks-ahead prediction and the predicted final size of the 2007 BTV-8 outbreak in Belgium based on the model fitted to various time points. The weekly cumulative number of infected cases in (A) is the prediction at 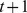 while (B) is the predicted cases at 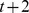, (C) on the other hand, is the predicted final outbreak size. Predictions are based on the model fitted to the 2006 outbreak data until week t of 2007. It was assumed that at the beginning of the 2007 outbreak, all farms are susceptible again.

To validate the performance of the model, the 2007 BTV outbreak was simulated using the model fitted to the 2006 data. Stochastic prediction results for simulations initialized using the observed 6 weeks data (761 cases of observed infection) are presented in Figure S2. The median stochastic final size of the outbreak was estimated to be 10 117 farms with 90% interval of 8 971–11 705, which was much higher than the observed 2007 outbreak size of 6 840 farms. In effect, the stochastic prediction estimated that around 25.2% of farms in Belgium would be infected by BTV at the end of 2007. However, this prediction assumes that the parameters underlying the two outbreaks are the same, hence it is not surprising that the model did not predict well the 2007 outbreak. Another way of performing model validation is to update the model with new observations and predict the values k-week (s) ahead. Hence, a one-week-ahead, two-weeks-ahead and final size prediction was preformed based on the model fitted not only to the 2006 data but also to the 2007 data at different weeks. Figures 10 A, B, and C show the performance of the model, where the one-week-ahead prediction is generally not far-off from the observed values, while the prediction two-weeks ahead of time is far from the observed value in some weeks. The final outbreak size prediction shows that the model prediction starts to stabilize from week 15 of the 2007 outbreak (using data from 2006 until week 15 of 2007 outbreak).

## Discussion

This paper presents a modelling framework for the transmission of BTV-8 across different farms in Belgium for 2006. Results from the model fit suggests that temperature, precipitation, farm density, land area, proportion of pasture and urban areas relative to crop area are important for describing the BT dynamics. Municipality-specific covariates explain the varying level of susceptibility of the different municipalities while the increase and decrease in transmission is explained by the temperature and precipitation values in the preceding 4 weeks. These results are not surprising given the fact that BTV is transmitted via a vector which thrives on certain climatic conditions, specifically, high temperature values [13] and high precipitation level [29], [30]. A risk factor which would have been interesting to include is the number of wind events during the outbreak. Hendrickx et al. [31] and Faes et al. [10] have found that wind was a significant contributor to the spread of the infection. But due to the unavailability of the data, it was left out of the model.

Fitting the spatio-temporal model also allowed the BTV-8 transmission process to be divided into different components: background, within-municipality, between-municipality, and movement transmission. The background transmission quantifies the inherent susceptibility of a municipality to bluetongue infection where the different covariate effects (except the intercept) does not depend on the previous infection status of the municipality. This measures the susceptibility of the municipalities at the beginning of the outbreak, and as such it is an important component of the model. The within-municipality transmission quantifies the susceptibility of municipalities to local spread of BTV given that infection has already been detected at the previous week in the municipality. The two possible routes for a municipality to contract BTV as stipulated in the model is through between-municipality transmission and movement of an infected animal. The between-municipality transmission quantifies the influence of the infection status of neighbouring municipalities (those with shared borders). This happens even though BTV-8 is non-contagious since presence of infection in neighbouring municipalities implies that a vector with the virus might be present, travel to the neighbouring municipality and may bite the animal in that municipality, causing the transmission of BTV. The neighbourhood assumption based on contiguous regions was deemed appropriate, given the fact that although *Culicoides* midges can be dispersed by the wind to great distances, dispersal over land follow a hopping pattern, i.e. with intermediary stops [31], and with the midges being able to fly only a maximum distance of 2 km [21]. The transmission through movements, on the other hand, quantifies the effect of animal transports in the transmission of BTV which allows us to study the effect of movement restrictions. Animal transport was quantified as a binary matrix weight signifying presence or absence of transport between municipalities. A constant general weight matrix was used. A weekly movement matrix was considered (data not shown) but offered no improvement over the constant assumption. The spatio-temporal model could be simplified by combining the within- and between-municipality components as one component representing the overall transmission by the vector thereby reducing the model components from four to three. However, as it is expected that *Culicoides* midges would cause more transmission in shorter distances (within-municipality) as compared to longer distances (between-municipality), we opted to keep the two components separate.

To investigate the effect of movement restriction on the spread of BTV, a one-step-ahead deterministic prediction and long-term stochastic prediction based on the model was performed with and without movement. For the one-step-ahead deterministic prediction, predicted weekly values were based on the previous week observed data and the parameter estimates from the model fitted to the whole data. The disadvantage of this procedure is the absence of uncertainty estimates especially for the weekly outbreak size. Stochastic prediction obtained by generating observations from a binomial distribution based on the predicted probabilities allowed the quantification of uncertainty for the predicted values. For this paper, parameter estimates from the model and data until the 7th week of outbreak were used as an initial condition and the model was allowed to predict the rest of the outbreak period. This coincides around the time period that the whole country of Belgium was declared as BTV-8 infected which resulted to the lifting of livestock transport restrictions [13].

Deterministic predictions showed a significant contribution of movement to the BTV outbreak at the end of 2006. Movement restriction would result to 200 fewer farms infected with BTV-8. Stochastic predictions also showed that movement restriction resulted only in local spreading and no infection in the West and East Flanders. This is an important result since it implies that movement of cattle caused the introduction of BTV-8 to these Flemish areas. De Koeijer et al. [13] in fact pointed out that the lifting of movement restrictions in Belgium resulted in long distance transmission and spatial pattern of transmission that was different from that of the Netherlands and Germany. Furthermore, simulating the effect of targeted restriction, specifically, restricting movement within a certain radius of the observed infection showed that it is effective in reducing the outbreak size. In fact, results have suggested that up to a certain radius around the infected farms, 10–15 km in this case, the movement restriction is as effective as the total ban all over Belgium in reducing the outbreak size. This was also observed by Turner et al. [14] for the BTV in England. This finding is important especially in guidelines formulation since a small restriction zone (e.g. 15 km) would lead to less adverse economic impact to the cattle industry than a larger zone (e.g. 70 km) or a total movement ban [32].

The model was validated with the 2007 BTV outbreak in Belgium. However, using the estimates from the model fitted to the 2006 outbreak gives a completely different predicted pattern. This might be due to the different nature of the outbreak in the two years. In 2007, BTV-8 was already present the year before and the model does not take this into account since it was built on the 2006 data before which infection had not previously occurred. Furthermore, reporting biases might have had an effect on the results and we did not take this into account. An ideal approach would have been to validate the model on data from other countries like the Netherlands and Germany which also experienced the BTV outbreak for the first time in 2006. Since these data from other countries are not available, a different validation approach was done, which was based on refitting the model to the new data each week and predicting one-week and two-weeks-ahead and the final size of the outbreak. Simulations show that the model performs well in terms of short-term prediction, but does not perform well in the long-term. However, as more data become available, the model was able to adapt to the new outbreak.

This study establishes the importance of movement restriction in reducing the outbreak size and preventing the long-distance transmission of BTV. This study also showed how to estimate different effects (local, neighbourhood and long-distance effects) in order to understand more what is happening during the outbreak. It also showed the importance of proper guidelines, especially in terms of the size of the restriction zones, in the reduction of outbreak size. It would be interesting to see an application of this model to other livestock diseases such as the recently discovered Schmallenberg virus [33] and to see the interplay of each component in the spreading of the virus.

## Supporting Information

Figure S1 **Spatial structure of the yearly total cattle purchases per municipality in Belgium for 2005–2009.**
(TIF)

Figure S2 **Prediction of the 2007 BTV-8 outbreak in Belgium based on the model fitted to the 2006 data.** The weekly number of predicted cases (A) and the cumulative number of cases (B and C) is based on 1000 stochastic predictions done using data until week 6 of the 2007 outbreak (July 01– August 05, 2007) and the model was then allowed to predict the outbreak until the end of 2007. The gray lines are the predictions from 1000 simulations.
(TIF)
