# Supplementary figures and images for: A Dynamic Spatio-Temporal Model to Investigate the Effect of Cattle Movements on the Spread of Bluetongue BTV-8 in Belgium
Source: PLoS One. 2013 Nov 11;8(11):e78591. doi: 10.1371/journal.pone.0078591 (PMC3823847; doi:10.1371/journal.pone.0078591)

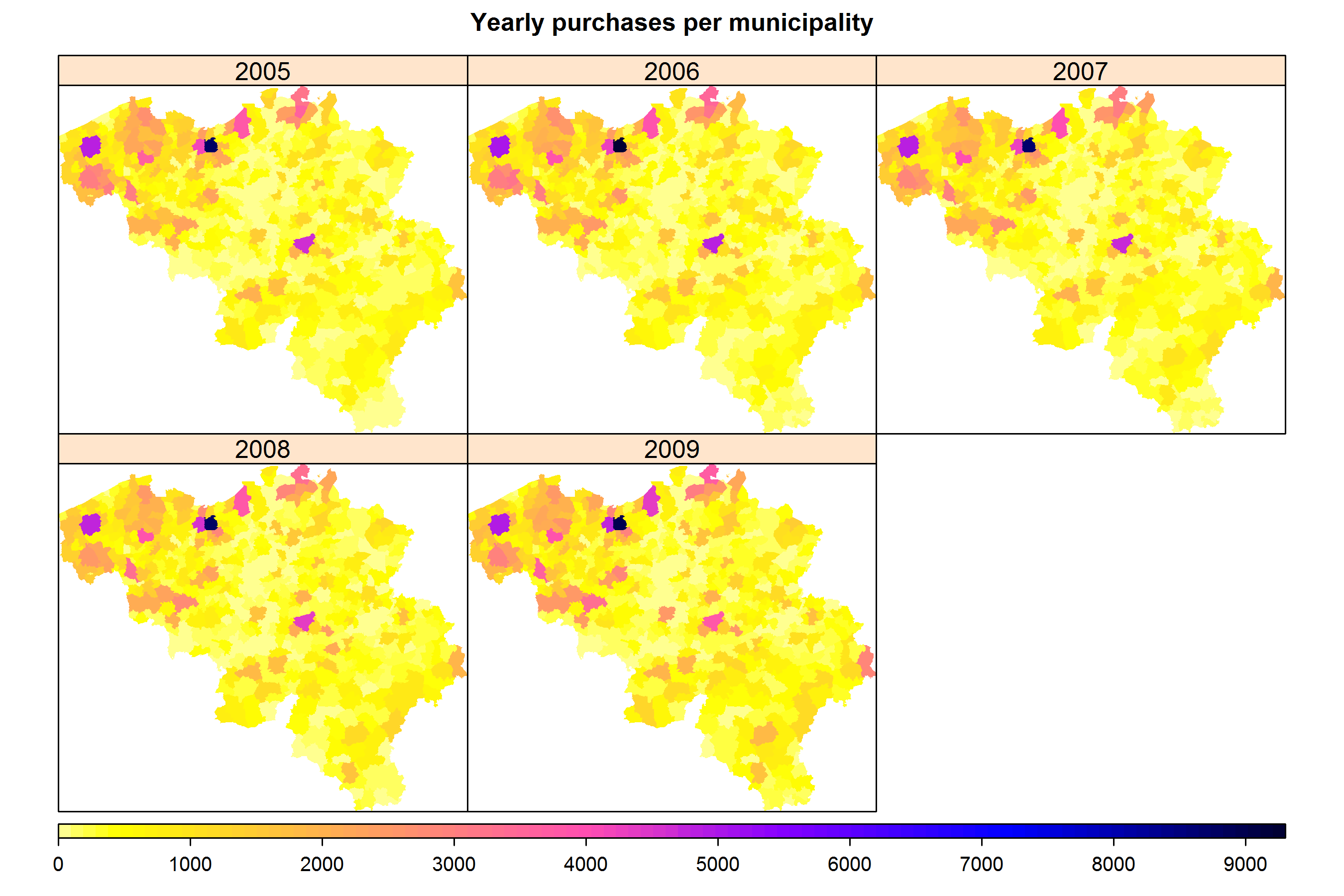

Supplement: Figure S1 — Spatial structure of the yearly total cattle purchases per municipality in Belgium for 2005–2009. (TIF) [file pone.0078591.s001.tif]

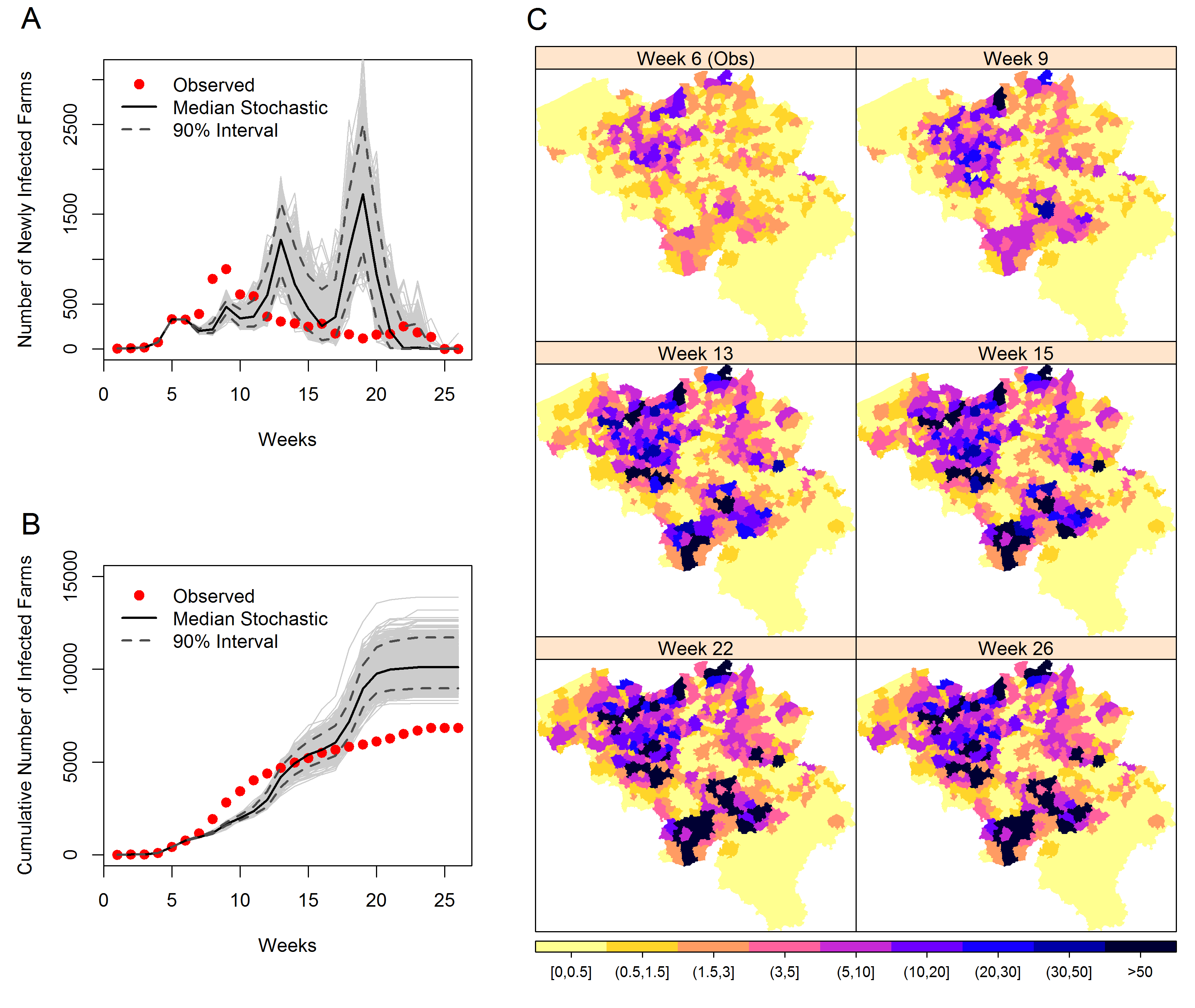

Supplement: Figure S2 — Prediction of the 2007 BTV-8 outbreak in Belgium based on the model fitted to the 2006 data. The weekly number of predicted cases (A) and the cumulative number of cases (B and C) is based on 1000 stochastic predictions done using data until week 6 of the 2007 outbreak (July 01– August 05, 2007) and the model was then allowed to predict the outbreak until the end of 2007. The gray lines are the predictions from 1000 simulations. (TIF) [file pone.0078591.s002.tif]
